# Circulating Exosomal SOCS2-AS1 Acts as a Novel Biomarker in Predicting the Diagnosis of Coronary Artery Disease

**DOI:** 10.1155/2020/9182091

**Published:** 2020-04-09

**Authors:** Caihong Liang, Lulu Zhang, Xiaoqing Lian, Tiantian Zhu, Yuqing Zhang, Ning Gu

**Affiliations:** ^1^Department of Cardiovasology, The Affiliated Jiangning Hospital with Nanjing Medical University, 168 Gushan Road, Nanjing, Jiangsu Province, China; ^2^Department of Endocrinology, The Affiliated Jiangning Hospital with Nanjing Medical University, 168 Gushan Road, Nanjing, Jiangsu Province, China; ^3^Department of Cardiovasology, Nanjing Hospital of Chinese Medicine Affiliated to Nanjing University of Chinese Medicine, 1 Jinling Road, Nanjing, Jiangsu Province, China

## Abstract

**Background and Aims:**

Critical roles of circulating exosomal long noncoding RNAs (lncRNAs) have been implicated in multiple diseases. However, little is known about their roles in coronary artery disease (CAD). The aim of the present study was to investigate the relationships between circulating exosomal lncRNAs and CAD and identify the aberrantly expressed disease-related lncRNAs as biomarkers in diagnosing CAD.

**Methods:**

The aberrantly expressed lncRNAs in plasma exosomes from CAD patients and controls were identified by microarray analysis and verified by quantitative real-time PCR (qRT-PCR). Then, the correlation between the expression level of candidate biomarker and clinic features in CAD patients, mild coronary artery stenosis (mCAS) patients, and controls was analyzed. Finally, we used the receiver operating characteristic (ROC) curve to examine the diagnosis value of candidate biomarkers.

**Results:**

The downregulated SOCS2-AS1 was determined by microarray analysis and verified by qRT-PCR in plasma from CAD patients in contrast to controls. The SOCS2-AS1 expression level in plasma exosomes was negatively correlated with PLT and Lpa. Moreover, CAD patients with elevated levels of plasma exosome-encapsulated SOCS2-AS1 were susceptible to multicoronary artery lesions. Additionally, the area under ROC (AUC) of SOCS2-AS1 was 0.704 (95% CI = 0.607–0.801, *P* < 0.001) for diagnosis of CAD.

**Conclusions:**

Plasma exosome-encapsulated SOCS2-AS1 was an independent protective factor against CAD and could be potentially used as a novel biomarker for the diagnosis of CAD.

## 1. Introduction

Coronary artery disease (CAD) is one of the leading causes of mortality in the world [1]. The pathogenesis of CAD remains unclear which are generally accepted as the results of the interaction between genetic and lifestyle factors [2]. As the main pathologic basis of CAD, atherosclerosis is a complex status with several pathological processes involving metabolism disorder, inflammation, and structural modification [3]. It has been generally established that atherosclerosis results from diverse cell dysfunctions and inflammatory processes affected, and these biological processes are regulated by the genome [4, 5]. Long noncoding RNAs (lncRNAs), one type of nonprotein-coding transcripts with more than 200 nucleotides in length, are important regulatory molecules in various pathological processes of multiple diseases [6]. Recently, numerous studies have indicated that lncRNAs, as important regulators, promote the development of atherosclerosis [7, 8]. Moreover, previous researchers have revealed several lncRNAs might be as potential biomarkers for CAD [9–11]. Exosomes, 40–100 nm diameter extracellular vesicles embedding proteins, lipids, and nucleic acids regulate lots of pathophysiological processes including metabolism, immune responses, and so on [12]. After being released into body fluids including plasma, urine, and saliva, exosomes could transport these biologically active molecules and regulate gene expression and cellular function in target cells [12]. It has been shown that the changes of exosome contents including lncRNAs can lead to significant alterations in certain biological processes, which may play important roles in the development and progression of CAD. Furthermore, exosome contents also can be measured as potential diagnostic and prognostic biomarkers and therapeutic targets for CAD [13, 14]. It was reported that exosomal lncRNA GAS5 could regulate the apoptosis of macrophages and vascular endothelial cells in atherosclerosis [15]. However, studies on the relationship between circulating exosomal lncRNAs and CAD remain sparse. Therefore, we conducted this microarray analysis of exosomal lncRNAs in plasma from CAD patients with angiography demonstrated in contrast to controls. The aim of the present study was to investigate the relationships between circulating exosomal lncRNAs and CAD and identify the aberrantly expressed disease-related lncRNAs as potential biomarkers in diagnosing CAD.

## 2. Materials and Methods

### 2.1. Patients

Study subjects were obtained from 276 patients who underwent coronary angiography for suspected or known CAD from September 2016 to September 2017 at the Affiliated Jiangning Hospital with Nanjing Medical University. The research protocol was approved by the Institutional Ethics Committee of the Affiliated Jiangning Hospital of Nanjing Medical University. Written informed consent was obtained from every participant of this study. Three cardiologists assessed the angiograms independently. CAD was defined if there was ≥50% luminal organic stenosis in one or more major coronary artery, including left anterior descending, left circumflex, and the right coronary artery. Subjects with <50% luminal organic stenosis in any major coronary artery were defined as mild coronary artery stenosis (mCAS) patients. In the present study, mCAS patients are considered as the early stage of CAD. Subjects with normal coronary arteries were considered as controls. Patients with severe concomitant diseases, including congenital heart disease, cardiomyopathy, hepatic failure, renal failure, bleeding disorders, and malignant tumor were excluded in this study. The relevant clinical data of all patients were available. Finally, 227 consecutive subjects were enrolled in this study and were divided into three groups according to the results of coronary angiograms: 111 patients in CAD group, 48 patients in mCAS group, and 68 patients in the control group.

Fasting peripheral blood samples of all subjects, including CAD patients, mCAS patients, and controls, were collected before coronary angiography in the morning. The samples were collected in a separate anticoagulated cube with ethylenediaminetetraacetic acid (EDTA), followed by centrifugation at 3,000 rpm for 10 min to retrieve plasma. All plasma samples were stored at −80°C until further analysis.

Clinical characteristics were also collected, including age, gender, smoking status, history of hypertension, history of diabetes, and the examinations of blood. Platelet (PLT) and C-reaction protein (CRP) levels were measured by a hematology analyzer (Beckman LH750, Beckman Coulter Co. Ltd., USA). Fibrinogen (FIB) and International Normalized Ratio (INR) were measured by a coagulation analyzer (Sysmex CA500, Sysmex Co. Ltd., Japan). Creatinine (Cr), Blood Urea Nitrogen (BUN), Fasting Blood Glucose (FBG), Uric Acid (UA), Total cholesterol (TC), Triglyceride (TG), High Density Lipoprotein-cholesterol (HDL-C), LDL-C (Low Density Lipoprotein-cholesterol), and Lipoprotein a (Lpa) were analyzed by biochemical analyzer (Beckman AU5800, Beckman Coulter Co. Ltd., USA).

### 2.2. Study Design

Three stages were carried out in looking for a biomarker or biomarkers, according to the following sequence: screening phase, training phase, and validation phase. Firstly, 3 CAD patients and 3 controls were selected by using a random number table, respectively, and then were used for microarray analysis in the screening phase. Next, 24 CAD patients and 24 controls were also selected by using a random number table, respectively, and were used in the training phase. 24 CAD patients and 24 controls were not paired samples. Finally, 48 mCAS patients, remaining 84 CAD patients and 41 controls were used in the validation phase.

In the screening phase, 3 CAD patients and 3 controls plasma exosome samples were selected randomly for microarray analysis (SHBIOCo. Ltd., China) to identify differently expressed lncRNAs in plasma exosome. Clinical characteristics of 3 CAD patients and 3 controls were presented in supplemental Table 1. LncRNAs with the most aberrant expression were selected as candidates for further study. In the training phase, all candidates were tested in independent cohort samples of 24 CAD patients and 24 controls. The expression levels of these candidates in CAD patients and controls were compared with quantitative real-time PCR (qRT-PCR). Candidates with significantly different expression levels were selected as potential biomarkers for further investigation. In the validation phase, the expression levels of potential biomarkers were detected in another independent cohort sample of 84 CAD patients and 41 controls. The biomarker was determined ultimately, based on these results. In order to clarify the expressions of this potential biomarker in an early stage of CAD, samples of 48 mCAS patients were detected.

Then, the relationship between the expression level of potential biomarker and clinic features was analyzed in study subjects. Moreover, we used receiver operating characteristic (ROC) curve to examine the diagnosis value of the potential biomarker. Finally, the diagnostic value of potential the biomarker was evaluated in an early stage of CAD.

### 2.3. Extraction of Exosomal RNAs

Plasma exosomes were isolated by Invitrogen™ Total Exosome Isolation Kit (Invitrogen, USA) following the manufacturer's instructions. Total RNA was extracted and purified using exoRNeasy Serum/Plasma Midi Kit (QIAGEN, Germany) according to the protocol. The quality of RNA samples was assessed by a UV spectrophotometer (Bio-Rad, USA), and the 260/280 nm absorbance ratio of samples was limited to 1.8–2.0. cDNAs were synthesized following the protocol of PrimeScriptTM RT reagent Kit (Takara, Japan) and then used as a template for qRT-PCR.

### 2.4. Quantitative Real-Time PCR (qRT-PCR)

Plasma exosomes-encapsulated lncRNAs were determined by qRT-PCR. qRT-PCR was carried out by using ABI Prism 7900HT (Applied Biosystems, USA) according to the manufacturer's instructions. The qRT-PCR reaction was performed according to the protocol of SYBR® Premix Ex Taq™ II (Takara, Japan). U6 snRNA and 5S rRNA were used as the internal control. The expression levels of exosomal lncRNAs were estimated by 2^–ΔΔCT^.

### 2.5. Statistical Analysis

All measurement variables were presented as mean ± standard deviation. The Student's *t*-test and Mann–Whitney unpaired test analysis were used to evaluate statistical differences between CAD patients and controls. Count data were analyzed by the chi-squared test. Analysis of variance (ANOVA) was used to compare multigroup variables. The area under the ROC curve (AUC) analysis was performed to estimate the effectiveness of the lncRNAs for prediction. Statistical analysis was performed by using SPSS 20.0 and GraphPad Prism 5.0. In all cases, *P* < 0.05 was considered significant. All *P* values were two-sided.

## 3. Results

### 3.1. Specific lncRNA Expressions in Plasma Exosomes from CAD Patients

**Figure 1:**
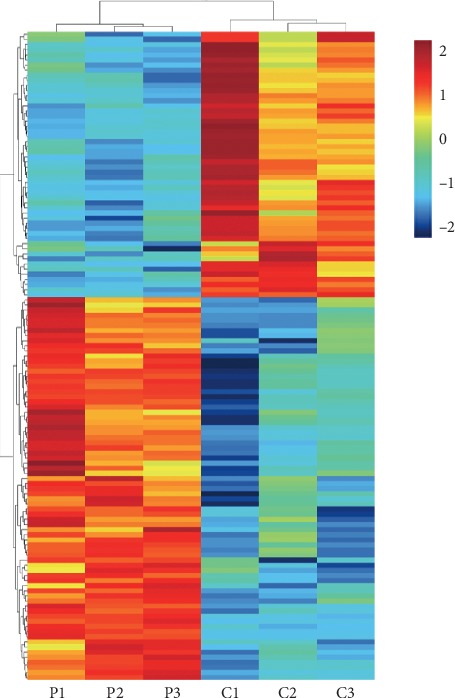
Hierarchical clustering analysis for specific lncRNAs (75 lncRNAs were upregulated and 52 lncRNAs were downregulated) in plasma exosomes from coronary artery disease (CAD) patients (criteria: fold change ≥2 or fold change ≤0.5; *P* < 0.05). P, Patient; C, Control.

**Figure 2:**
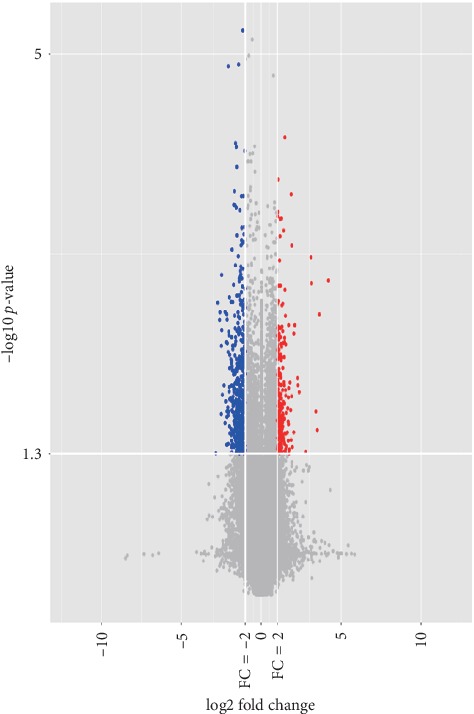
Volcano plot analysis of the microarray chip data on the differentially expressed plasma exosomal lncRNAs between coronary artery disease (CAD) patients and controls. The vertical white line corresponds to a 2.0-fold up- and downregulation while the horizontal white line represents a *P* value of 0.05. The red and blue dots indicate more than a 2.0-fold change and represent the differentially expressed genes with statistical significance.

A total of 127 aberrantly expressed lncRNAs (75lncRNAs were upregulated and 52 lncRNAs were downregulated) in plasma exosomes from CAD patients were identified by microarray analysis (criteria: fold change ≥2 or fold change ≤0.5; *P* < 0.05) (Figures 1, 2). In order to further narrow down the number of candidates, we selected transcripts with an average normalized intensity higher than 7. Among 16 candidates, 3lncRNAs were upregulated and13 lncRNAs were downregulated (Table 1).

### 3.2. Expressions of Exosomal LncRNA Candidates in CAD Patients Compared with Controls in the Training Phase and Validation Phases

**Figure 3:**
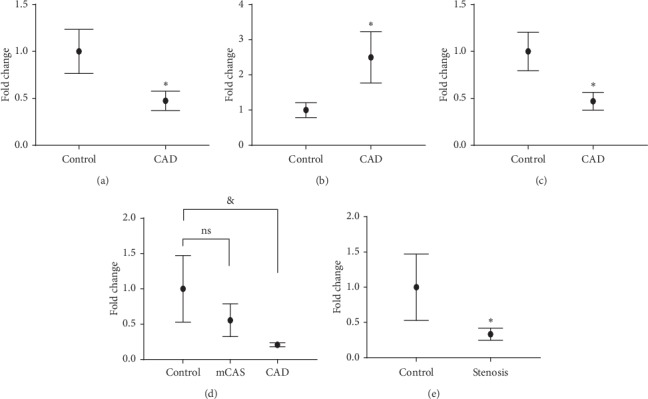
Validation of differentially expressed plasma exosomal miRNAs by quantitative real-time PCR (qRT-PCR). (a) Relative expression of AC017053.1 in plasma exosomes from 24 CAD patients and 24 controls. (b) Relative expression of NONHSAT138339 in plasma exosomes from 24 CAD patients and 24 controls. (c) Relative expression of SOCS2-AS1 in plasma exosomes from 24 CAD patients and 24 controls. (d) Relative expression of SOCS2-AS1 in plasma exosomes from 84 CAD patients, 48 mCAS patients, and 41 controls. (e) Relative expression of SOCS2-AS1 in plasma exosomes from the stenosis group (merged 84 CAD patients and 48 mCAS patients) and 41 controls. ^*∗*^*P* < 0.05; & *P* < 0.01.

As a result, lower expression levels of SOCS2-AS1 and AC017053.1 and higher expression levels of NONHSAT138339 were observed in plasma exosomes of CAD patients in the training phase (Figures 3(a)–3(c)). In the validation phases, we verified that exosomal SOCS2-AS1 was downregulated obviously, consistent with the result in the training phase in CAD patients compared with controls (Figure 3(d)). However, no statistical significance was shown for the expression levels of AC017053.1 and NONHSAT138339 in CAD patients and controls.

### 3.3. Correlation between Plasma Exosome-Encapsulated SOCS2-AS1 Levels with Clinical Characteristics

The clinical characteristics of CAD patients and controls were presented in Supplemental Table 2. Compared with controls, CAD patients had a higher percentage of elderly people and smokers. Additionally, higher levels of CRP, FIB, INR, BUN, UA but a lower level of HDL-C were also found in CAD patients.

The correlations of plasma exosome-encapsulated SOCS2-AS1 level with clinical characteristics in CAD patients were analyzed by Pearson correlation. As shown in Table 2, plasma exosome-encapsulated SOCS2-AS1 level was negatively associated with Lpa and PLT. Partial correlation analyses that control for all potentially confounding variables, including age, gender, BMI, smoking, hypertension, diabetes, FBG, BUN, Cr, UA, CRP, FIB, INR, TC, TG, HDL-C, and LDL-C, were further carried out to estimate the stability of the correlations. As a result, plasma exosome-encapsulated SOCS2-AS1 level was also negatively associated with Lpa and PLT after controlling all potentially confounding variables (Table 2, model 1–3).

### 3.4. Plasma Exosome-Encapsulated SOCS2-AS1 Is the Independent Protective Factor against CAD

As presented in Table 3, plasma exosome-encapsulated SOCS2-AS1 level was certified as the independent protective factor for CAD by univariate logistic regression analysis (OR = 0.328, 95% CI = 0.173–0.623, *P*=0.001). Even after adjustment for age, gender, smoking, hypertension, diabetes, FBG, BUN, Cr, UA, CRP, PLT, FIB, INR, TC, TG, HDL-C, LDL-C and Lpa, multivariate logistic regression analysis also revealed that plasma exosome-encapsulated SOCS2-AS1 level was the independent protective factor for CAD (Table 3, model^1^–model^6^).

Due to differences in some clinical characteristics between CAD patients and controls, stratification analyses were further carried out to assess any effect modification by these potentially confounding factors. Examinations of blood, such as CRP, FIB, INR, BUN, UA, and HDL-C, were divided into different subgroups according to normal reference values. As shown in Supplemental Table 3, plasma exosome-encapsulated SOCS2-AS1 level was the protective factor for CAD in all subgroups stratified by age, gender, FIB, INR, BUN, and UA except the subgroups that could not be analyzed by software due to small sample size. Statistical analysis showed no significant differences in the subgroup of higher CRP levels and lower HDL-C levels.

### 3.5. The Clinical Relevance Analysis of Plasma Exosome-Encapsulated SOCS2-AS1 Level in CAD Patients

On the basis of coronary arteriography examination results, 84 CAD patients were divided into single coronary artery lesion subgroup and multicoronary artery lesions subgroup according to lesion artery numbers, and into severe subgroup (≥75% luminal stenosis of any coronary vessel) and slight subgroup (50% ≤ luminal stenosis of any coronary vessel <75%) according to luminal stenosis degree of coronary arteries. The mean value of SOCS2-AS1 levels in controls was set as the cut-off value to classify the SOCS2-AS1 expression levels of CAD patients. All 84 CAD patients weredivided into lower expression group and higher expression group according to different grouping criteria. The measurement data were mean ± standard deviation for statistical description. As shown in Table 4, the clinical relevance analysis of CAD patients demonstrated that a higher expression level of exosomal SOCS2-AS1 in plasma was correlated with lower levels of PLT.

### 3.6. ROC Curve Analysis for Predicting the Diagnosis of CAD

**Figure 4:**
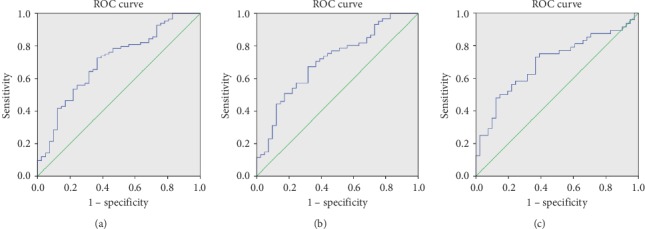
The receiver operating characteristic (ROC) curve analyses for plasma exosomal SOCS2-AS1 as a diagnostic biomarker of CAD. (a) The area under ROC curve (AUC) was 0.704 (95% CI = 0.607–0.801, *P* < 0.001) for diagnosis of CAD patients. The sensitivity and specificity at the optimal cut-off were 71.4% and 63.4%. (b) The AUC was 0.709 (95% CI = 0.607–0.811, *P* < 0.001) for diagnosing severe subgroup of CAD patients (≥75% luminal stenosis of any coronary vessel). The sensitivity and specificity at the optimal cut-off were 67.2% and 68.3%. (c) The AUC was 0.698 (95% CI = 0.588–0.807, *P*=0.001) for diagnosis of mild coronary artery stenosis (mCAS) patients (50% ≥ luminal stenosis of any coronary vessel >0). The sensitivity and specificity at the optimal cut-off were 72.9% and 63.4%.

The area under ROC curve (AUC) was 0.704 (95% CI = 0.607–0.801, *P* < 0.001) for diagnosis of CAD patients and was 0.709 (95% CI = 0.607–0.811, *P* < 0.001) for diagnosing severe subgroup of CAD patients (Figures 4(a), 4(b)). The sensitivity and specificity at the optimal cut-off were 71.4% and 63.4% for diagnosing CAD patients, and 67.2% and 68.3% for diagnosing severe subgroup of CAD patients.

### 3.7. Expressions of Plasma Exosome-Encapsulated SOCS2-AS1 and Clinical Characteristics in mCAS Patients

As shown in Figure 3(d), we found SOCS2-AS1 was downregulated in mCAS patients, as an early stage of CAD, compared with controls; however, the result was not statistically significant. Meanwhile, a trend that the level of exosomal SOCS2-AS1 was inversely associated with the severity of atherosclerosis was also shown after comparing the expression levels of CAD patients, mCAS patients, and controls, although the result was not statistically significant. Therefore, we merged mCAS patients and CAD patients as one group (stenosis group), and then compared the SOCS2-AS1 expression between the stenosis group and controls. As shown in Figure 3(e), the expressions of SOCS2-AS1 in the stenosis group were lower than controls.

The clinical characteristics of mCAS patients, as an early stage of CAD, were also presented in Supplemental Table 4. The levels of INR and BUN in mCAS patients were higher as compared with controls. Besides, elderly people were more likely to suffer from mCAS.

### 3.8. Diagnostic Value of Plasma Exosome-Encapsulated SOCS2-AS1 in an Early Stage of CAD

ROC curve analysis was also carried out for evaluating the diagnostic value of plasma exosome-encapsulated SOCS2-AS1 in the early stage of CAD. The AUC was 0.698 (95% CI = 0.588–0.807, *P*=0.001) for diagnosis of mCAS patients, and the sensitivity and specificity at the optimal cut-off were 72.9% and 63.4% (Figure 4(c)).

As shown in Supplemental Table 5, plasma exosome-encapsulated SOCS2-AS1 level was certified as the independent protective factor for mCAS by univariate logistic regression analysis (OR = 0.438, 95% CI = 0.253–0.758, *P*=0.003). Even after adjustment for age, gender, smoking, hypertension, diabetes, FBG, BUN, Cr, UA, CRP, PLT, FIB, INR, TC, TG, HDL-C, LDL-C, and Lpa, multivariate logistic regression analysis also revealed that plasma exosome-encapsulated SOCS2-AS1 level was the independent protective factor for mCAS (Supplemental Table 5, model^1^–model^6^).

## 4. Discussion

CAD continues to be major cardiovascular mortality and morbidity worldwide. Despite great advances in recent years, diagnosing CAD with noninvasive methods remains difficult, especially in its early stage. Several previous reports presented that lncRNAs might be potential biomarkers for CAD [9–11]. Currently, it has been recognized that circulating exosomes released from leukocytes, platelets, and endothelial cells, as regulators of CAD, could reflect the stage or progression of the disease [16]. It has also been proved that the changes of exosome contents including lncRNAs may be measured as potential diagnostic and prognostic biomarkers and therapeutic targets for CAD [13, 14, 17]. Therefore, we aimed to determine whether or not certain circulating exosome-encapsulated lncRNAs could be as promising biomarkers for the diagnosis of CAD, especially in an early stage.

In the present study, we compared the lncRNA expression profiles in plasma exosomes between CAD patients and controls by microarray screening and validated the results by qRT-PCR. As a result, we verified that plasma exosome-encapsulated SOCS2-AS1 level was downregulated obviously in CAD patients compared with controls. Moreover, we clarified that plasma exosome-encapsulated SOCS2-AS1 level was the independent protective factor against CAD. We also detected the expressions of SOCS2-AS1 in mCAS patients, an early stage of CAD. After comparing the expression level of SOCS2-AS1 between mCAS patients and controls, no statistically significant was shown. However, a trend that the level of exosomal SOCS2-AS1 was inversely associated with the severity of atherosclerosis was also shown after comparing the expression levels of CAD patients, mCAS patients, and controls. It is accepted that the development of coronary artery stenosis is a gradual process 50% luminal organic stenosis in the major coronary artery was only used as the definition of CAD subjectively. Therefore, we merged mCAS patients and CAD patients as one group (stenosis group), and then compared the SOCS2-AS1 expression between the stenosis group and controls. As shown above, the expressions of SOCS2-AS1 in the stenosis group were lower than controls. Moreover, plasma exosome-encapsulated SOCS2-AS1 level was also certified as the protective factor for mCAS which was similar to CAD. Apparently, these findings suggest that mCAS, with the same pathogenesis and similar SOCS2-AS1 expressions, is the different stage of CAD and is different from controls. Small sample size may lead to the liable of no statistical significance in the expression level of SOCS2-AS1 between mCAS patients and controls.

Subsequently, it was shown that SOCS2-AS1 could be a novel biomarker for the diagnosis of CAD by ROC curve analysis. Meanwhile, ROC curve analysis indicated that SOCS2-AS1 also could be a fine biomarker with a higher sensitivity for diagnosing mCAS. Although with relatively higher sensitivity and specificity than previous homogeneous biomarker [9], as a downregulated biomarker, SOCS2-AS1, with both sensitivity and specificity less than 80%, is not an ideal diagnostic biomarker. However, it is very important that SOCS2-AS1 is a novel potential diagnostic biomarker for CAD, especially for an early stage of CAD. Although in the current stage, there are still some shortcomings, it might be improved later by further studies.

In the present study, univariate and multivariate logistic regression analyses showed that SOCS2-AS1 was the independent protective factor against CAD. However, due to differences in some clinical characteristics between CAD patients and controls, stratification analyses were further carried out to assess any effect modification by these potentially confounding factors. As shown above, statistical analysis only showed no significant differences in the subgroup of higher CRP level and lower HDL-C level, which might be related to the small samples. SOCS2-AS1 was the protective factor for CAD in almost all subgroups stratified by every potentially confounding factor, which suggested that SOCS2-AS1 was the independent protective factor against CAD.

In the following research, we further found that plasma exosome-encapsulated SOCS2-AS1 level was negatively associated with Lpa and PLT even after controlling for all potentially confounding variables, suggesting that SOCS2-AS1 might be closely related to lipid metabolism and PLT. Meanwhile, the clinical relevance analysis of CAD patients also demonstrated that the SOCS2-AS1 expression level in plasma exosome was also correlated with PLT, strengthening the evidence linking SOCS2-AS1 with lipid metabolism and PLT. These findings suggested that SOCS2-AS1 might be closely related to atherosclerosis.

LncRNAs serve as versatile regulators of various aspects of biological and pathological processes [18, 19], and accumulating evidence suggests that lncRNAs play crucial roles in the progress of atherosclerosis, the main pathologic basis of CAD, by regulating lipid metabolism [20, 21]. We hypothesized that SOCS2-AS1 might be connected tightly with lipid metabolism. Potential mechanisms of SOCS2-AS1 in lipid metabolism have not been confirmed. However, as a member of divergent lncRNAs family, SOCS2-AS1 may play similar roles. Divergent lncRNAs, comprising almost 20% of total lncRNAs, are transcribed in the opposite direction to nearby protein-coding genes [22]. Although the modulating functions and mechanisms of divergent lncRNAs remain largely unknown, positive regulations on nearby transcription by this class of transcripts were observed in several previous studies. A previous study clarified the mechanism of Evx1, a divergent lncRNA, as mediated coding gene Evx1 cis-regulation [23]. The researchers further identified that 75% of divergent lncRNAs led to the downregulation of nearby coding genes after being silenced in mouse and human [23]. As a prevalent phenomenon, SOCS2 might be regulated by SOCS2-AS1.

Abnormal lipid metabolism plays an important role in the development of atherosclerosis. Aberrant regulation of immune cells on lipid metabolism partly explains the underlying mechanism [24]. It has been clarified that SOCS2, as an important regulator of inflammation, is vital for maintaining immune homeostasis [25]. The overexpression of SOCS2 could decrease the expression of inflammatory cytokines [26, 27]. SOCS2 knockout or deficiency led to increasing nuclear factor *κ*B (NF-*κ*B) activity as well as elevated expression of genes for the inflammatory cytokines [25, 28]. A key aspect of atherosclerosis is the maladaptive inflammatory response to lipid accumulation in the artery and NF-*κ*B activation as a pathological mechanism of lipid metabolism and atherosclerosis [29]. Our previous study also demonstrated that NF-*κ*B pathway played vital roles in atherosclerosis via promoting oxidized low density lipoproteins induced oxidative stress and cell apoptosis in macrophages [30]. In humans, divergent lncRNAs, neighboring protein-coding genes were strongly enriched in nuclear functions, including transcription factor activity, sequence-specific DNA binding, and embryo development [22]. Thus, SOCS2-AS1 might play vital roles in atherosclerosis via regulating NF-*κ*B pathway through the cis-regulation of SOCS2. However, the exact mechanism needs further exploration.

Interestingly, SOCS2-AS1 is also closely related to PLT. Recent research pointed to the role of PLT derived exosomes in mediating platelet atherogenic interactions with endothelial cells and monocytes [31, 32]. It was demonstrated that endothelial cells could enhance their adhesiveness by uptaking of PLT derived exosomes, which was important in the pathogenesis of atherosclerosis [33, 34]. Meanwhile, PLT derived exosomes also could improve the activation of monocytes and monocyte [35].

Some limitations must be considered in our study. First of all, as a single center, small sample size and retrospective study, various forms of bias in our study might be liable to inaccurate conclusions. In the present study, there is no statistical significance in the expression level of SOCS2-AS1 between mCAS patients and controls which might be due to small sample size. Therefore, a large-scale, multicenter, and prospective validation study would be carried out in the near future. Secondly, the underlining mechanisms of the association between SOCS2-AS1, as an independent protective factor, and the CAD are not clear. The specific mechanisms should be explored in further experimental research. Last but not the least, SOCS2-AS1, with relatively high sensitivity and specificity, is far away from an excellent biomarker. With relatively high sensitivity and specificity than previous homogeneous biomarker and an advantage in the diagnosis of the early stage of CAD, SOCS2-AS1 bids fair to become a novel potential diagnostic biomarker for CAD. Although in the current stage, there are still some shortcomings, it might be improved later by further studies. To improve the diagnostic value, a combination of SOCS2-AS1 and other biomarkers would be analyzed in our subsequent study.

Notwithstanding some of the aforementioned limitations, our study suggests that plasma exosomes-derived SOCS2-AS1 is a potential biomarker for CAD. Although the underlying mechanisms remain largely unknown, the association between plasma exosomal SOCS2-AS1 and CAD is well documented. Future studies with high quality are warranted for further investigation.

## Figures and Tables

**Table 1:** The expression profile of lncRNA candidates in plasma exosomes of CAD patients compared with controls by microarray.

LncRNAs	Fold change	*P* value	Regulation
Lnc-USP9Y-16 : 1	0.476985	0.000144	Down
Lnc-STXBP6-5 : 1	0.451161	0.000812	Down
Lnc-ATG2B-3 : 2	0.452216	0.004192	Down
Lnc-PIGP-5 : 2	2.138978	0.006523	Up
Lnc-C22orf34-8 : 2	2.500921	0.007907	Up
AC017053.1	0.46897	0.008938	Down
NONHSAT096303	0.436393	0.009256	Down
SOCS2-AS1	0.452054	0.014885	Down
Lnc-TM2D2-2 : 1	0.402779	0.017852	Down
Lnc-SOX1-4 : 6	0.432825	0.02561	Down
RP11-527J8.1	0.494858	0.02578	Down
Lnc-MRPL10-1 : 2	0.491939	0.028083	Down
RP4-781K5.4	0.463193	0.030257	Down
Lnc-NAPEPLD-5 : 6	0.413648	0.030533	Down
NONHSAT120351	0.478036	0.03698	Down
NONHSAT138339	2.255479	0.040974	Up

**Table 2:** Correlations between plasma exosome-encapsulated SOCS2-AS1 level with clinical characteristics.

Variables	Pearson correlation		Partial correlation					
	*R*	*P* value	Model 1		Model 2		Model 3	
			*R*	*P* value	*R*	*P* value	*R*	*P* value
PLT	−0.166	0.029					−0.186	0.020
Lpa	−0.184	0.016	−0.181	0.022	−0.168	0.036		
TC	−0.037	0.627	−0.015	0.855				
TG	−0.046	0.548	−0.054	0.496				
HDL-C	0.055	0.471	0.025	0.758				
LDL-C	−0.072	0.348	−0.031	0.698				
Age	−0.145	0.057						
Gender	−0.025	0.748						
Smoking	−0.076	0.322						
Hypertension	−0.127	0.097						
FBG	−0.027	0.722						
Diabetes	−0.051	0.506						
BUN	−0.024	0.753						
Cr	−0.040	0.602						
UA	−0.142	0.063						
FIB	−0.022	0.766						
INR	−0.046	0.547						
CRP	−0.049	0.518						

CRP: C-reaction protein; PLT: Platelet; FIB, Fibrinogen; INR: International Normalized Ratio; BUN, Blood Urea Nitrogen; Cr: Creatinine; FBG: Fasting Blood Glucose; UA: Uric Acid; TC, Total Cholesterol; TG: Triglyceride; HDL-C: High Density Lipoprotein-cholesterol; LDL-C: Low Density Lipoprotein-cholesterol; Lpa: Lipoprotein a. The model 1 control Age, Gender, Smoking, Hypertension, Diabetes, FBG, BUN, Cr, UA, FIB, INR, CRP, and PLT; The model 2 control Age, Gender, Smoking, Hypertension, Diabetes, FBG, BUN, Cr, UA, FIB, INR, CRP, TC, TG, HDL-C, LDL-C, and PLT; The model 3 control Age, Gender, Smoking, Hypertension, Diabetes, FBG, BUN, Cr, UA, FIB, INR, CRP, TC, TG, HDL-C, LDL-C, and Lpa. ^*∗*^*P* < 0.05.

**Table 3:** Univariate analysis and multiple logistic regression analysis for the risk of CAD.

Models	OR	95% CI	*P* value
Univariate analysis	0.328	0.173–0.623	0.001^&^
Multiple logistic regression model^1^	0.350	0.175–0.701	0.003^&^
Multiple logistic regression model^2^	0.352	0.173–0.714	0.004^&^
Multiple logistic regression model^3^	0.337	0.163–0.697	0.003^&^
Multiple logistic regression model^4^	0.335	0.157–0.714	0.005^&^
Multiple logistic regression model^5^	0.323	0.146–0.717	0.005^&^
Multiple logistic regression model^6^	0.291	0.116–0.731	0.009^&^

OR: odds ratio; CI: confidence interval. The model^1^ included age, gender, smoking, and SOCS2-AS1 level; The model^2^ included age, gender, smoking, hypertension, and SOCS2-AS1 level; The model^3^ included age, gender, smoking, hypertension, diabetes, FBG, and SOCS2-AS1 level; The model^4^ included age, gender, smoking, hypertension, diabetes, FBG, BUN, Cr, UA, and SOCS2-AS1 level; The model^5^ included age, gender, smoking, hypertension, diabetes, FBG, BUN, Cr, UA, CRP, PLT, FIB, INR and SOCS2-AS1 level; The model^6^ included age, gender, smoking, hypertension, diabetes, FBG, BUN, Cr, UA, CRP, PLT, FIB, INR, TC, TG, HDL-C, LDL-C, Lpa, and SOCS2-AS1 level. ^&^*P* < 0.01.

**Table 4:** The clinical relevance analysis of SOCS2-AS1 expression levels in CAD patients.

Feather	Lower expression (*n* = 62)	Higher expression (*n* = 22)	*P* value
Age (years)			0.778
≤60	15	4	
>60	47	18	

Gender			0.176
Male	38	17	
Female	24	5	

Smoking			0.904
Yes	12	4	
No	50	18	

Hypertension			0.622
Yes	43	14	
No	19	8	

Diabetes			0.861
Yes	21	7	
No	41	15	

Lesion artery number			0.154
Single	23	12	
Multi	39	10	

Severity of artery stenosis			0.569
Severe	44	17	
Slight	18	5	
CRP (mg/L)	13.87 ± 28.43	9.93 ± 15.45	0.538
PLT (10^9^/L)	182.37 ± 50.77	141.50 ± 47.39	0.001^&^
FIB (g/L)	2.63 ± 0.92	2.43 ± 0.86	0.384
INR	1.04 ± 0.76	1.08 ± 0.78	0.053
BUN (mmol/L)	5.95 ± 2.15	6.51 ± 2.68	0.331
Cr (*μ*mol/L)	83.22 ± 47.97	116.94 ± 159.77	0.340
FBG (mmol/L)	6.44 ± 2.10	7.71 ± 4.02	0.169
UA (*μ*mol/L)	350.92 ± 79.42	341.82 ± 88.51	0.655
TC (mmol/L)	4.34 ± 1.05	4.06 ± 1.02	0.275
TG (mmol/L)	2.13 ± 2.21	1.83 ± 1.10	0.544
HDL-C (mmol/L)	1.27 ± 0.30	1.25 ± 0.25	0.793
LDL-C (mmol/L)	2.31 ± 0.74	2.11 ± 0.74	0.292
Lpa (mg/L)	267.75 ± 228.25	179.85 ± 275.71	0.146

CRP: C-reaction protein; PLT: Platelet; FIB: Fibrinogen; INR: International Normalized Ratio; BUN: Blood Urea Nitrogen; Cr: Creatinine; FBG: Fasting Blood Glucose; UA: Uric Acid; TC: Total Cholesterol; TG: Triglyceride; HDL-C: High Density Lipoprotein-cholesterol; LDL-C: Low Density Lipoprotein-cholesterol; Lpa: Lipoprotein a. ^&^*P* < 0.01.

## Data Availability

The data used to support the findings of this study are available from the corresponding author upon reasonable request.

## Supplementary Materials

Supplementary Materials Supplemental Table 1: clinical characteristics of CAD patients and controls. Supplemental Table 2: clinical characteristics of CAD patients and controls. Supplemental Table 3: stratification analyses of plasma exosome-encapsulated SOCS2-AS1 level with the risk of CAD. Supplemental Table 4: clinical characteristics of mCAS patients and controls. Supplemental Table 5: univariate analysis and multiple logistic regression analysis for the risk of mCAS.
