# Supplementary material for: Circulating Exosomal SOCS2-AS1 Acts as a Novel Biomarker in Predicting the Diagnosis of Coronary Artery Disease
Source: Biomed Res Int. 2020 Apr 9;2020:9182091. doi: 10.1155/2020/9182091 (PMC7171639; doi:10.1155/2020/9182091)
Supplement: Supplementary Materials — Supplemental Table 1: clinical characteristics of CAD patients and controls. Supplemental Table 2: clinical characteristics of CAD patients and controls. Supplemental Table 3: stratification analyses of plasma exosome-encapsulated SOCS2-AS1 level with the risk of CAD. Supplemental Table 4: clinical characteristics of mCAS patients and controls. Supplemental Table 5: univariate analysis and multiple logistic regression analysis for the risk of mCAS. [file 9182091.f1.pdf]

Supplemental Table 1. Clinical characteristics of CAD patients and controls.

Characteristics	Patient 1	Patient 2	Patient 3	Control 1	Control 2	Control 3
Age(years)	63	77	44	67	63	59
Gender	Female	Male	Male	Male	Male	Female
Smoking	No	No	Yes	Yes	No	No
Hypertension	Yes	Yes	No	No	Yes	No
Diabetes	Yes	Yes	No	No	Yes	No
Lesion Artery Number	Multi	Single	Multi	No	No	No
Severity of Artery Stenosis	Severe	Slight	Severe	No	No	No

Severe: ≥75% luminal stenosis of any coronary vessel; Slight: 50%≤luminal stenosis of any coronary vessel<75%.

Supplemental Table 2. Clinical characteristics of CAD patients and controls.

**Characteristics**	**Control(n=41)**	**CAD(n=84)**	***P* value**
Age(years)			0.001[&]
≤60	22	19	
>60	19	65	
Gender			0.125
Male	21	55	
Female	20	29	
Smoking			0.034*
Yes	2	16	
No	39	68	
Hypertension			0.071
Yes	21	57	
No	20	27	
Diabetes			0.856
Yes	13	28	
No	28	56	
CRP(mg/L)	3.93±12.43	12.84±25.65	0.010*
PLT(10^9/L)	166.20±46.05	171.67±52.81	0.572
FIB(g/L)	2.18±0.51	2.58±0.91	0.002[&]
INR	1.02±0.07	1.05±0.08	0.015*
BUN (mmol/L)	4.99±1.59	6.10±2.29	0.007[&]
Cr (μmol/L)	71.26±20.80	92.05±91.50	0.154
FBG (mmol/L)	6.56±2.92	6.77±2.77	0.692
UA (μmol/L)	310.51±87.15	348.54±81.45	0.018*
TC (mmol/L)	4.03±0.95	4.27±1.04	0.228
TG (mmol/L)	1.68±0.84	2.05±1.97	0.255
HDL-C (mmol/L)	1.38±0.26	1.27±0.29	0.033*

LDL-C (mmol/L)	2.05±0.66	2.25±0.74	0.135
Lpa(mg/L)	240.08±215.54	244.73±242.96	0.917

CAD, Coronary Artery Disease; CRP, C-reaction protein; PLT, Platelet; FIB, Fibrinogen; INR, International Normalized Ratio; BUN, Blood Urea Nitrogen; Cr, Creatinine; FBG,Fasting Blood Glucose; UA, Uric Acid; TC,Total Cholesterol; TG, Triglyceride; HDL-C, High Density Lipoprotein- cholesterol; LDL-C, Low Density Lipoprotein-cholesterol; Lpa, Lipoprotein a.

* $P < 0.05$, & $P < 0.01$.

Supplemental Table 3. Stratification analyses of plasma exosome-encapsulated SOCS2-AS1 level with the risk of CAD.

**Subgroup**			***OR***	***95%CI***	***P* value**
Age (years)					
	≤60	(n=41)	0.327	0.111-0.961	0.042*
	＞60	(n=84)	0.348	0.146-0.828	0.017*
Gender					
	Male	(n=76)	0.334	0.148-0.753	0.008$^{\&}$
	Female	(n=49)	0.279	0.089-0.873	0.028*
CRP (mg/L)					
	≤10	(n=105)	0.322	0.165-0.629	0.001$^{\&}$
	＞10	(n=20)	0.167	0.001-24.592	0.482
FIB(g/L)					
	＜2.0	(n=35)	0.248	0.063-0.982	0.047*
	2.0-4.0	(n=82)	0.382	0.185-0.787	0.009$^{\&}$
	＞4.0	(n=8)	Null	Null	Null
INR					
	＜0.8	(n=0)	Null	Null	Null
	0.8-1.2	(n=121)	0.339	0.179-0.642	0.001$^{\&}$
	＞1.2	(n=4)	Null	Null	Null
BUN(mmol/L)					
	＜3.1	(n=9)	Null	Null	Null
	3.1-8	(n=102)	0.338	0.174-0.660	0.001$^{\&}$
	＞8	(n=14)	Null	Null	Null
UA(μmol/L)					
	＜214	(n=6)	Null	Null	Null
	214-688	(n=113)	0.353	0.181-0.688	0.002$^{\&}$
	＞688	(n=6)	Null	Null	Null
HDL-C (mmol/L)					
	＜1.04	(n=24)	0.168	0.022-1.278	0.085
	1.04-1.40	(n=56)	0.271	0.083-0.883	0.030*
	＞1.40	(n=45)	0.421	0.185-0.958	0.039*

OR, odds ratio; CI, confidence interval. CRP, C-reaction protein; FIB, Fibrinogen;

INR, International Normalized Ratio; BUN, Blood Urea Nitrogen; UA, Uric Acid; HDL-C, High Density Lipoprotein- cholesterol. Null, sample size is too small to analyze.

* $P < 0.05$, & $P < 0.01$.

Supplemental Table 4. Clinical characteristics of **mCAS** patients and controls.

**Characteristics**	**Control(n=41)**	**mCAS (n=48)**	***P* value**
Age(years)			0.011*
≤60	22	13	
＞60	19	35	
Gender			0.271
Male	21	19	
Female	20	29	
Smoking			0.210
Yes	2	6	
No	39	42	
Hypertension			0.501
Yes	21	28	
No	20	20	
Diabetes			0.483
Yes	13	12	
No	28	36	
CRP(mg/L)	3.93±12.43	3.80±9.67	0.955
PLT(10^9/L)	166.20±46.05	160.40±51.23	0.579
FIB(g/L)	2.18±0.51	2.20±0.48	0.829
INR	1.02±0.07	1.05±0.09	0.040*
BUN (mmol/L)	4.99±1.59	5.74±1.79	0.043*
Cr (μmol/L)	71.26±20.80	67.81±13.23	0.363
FBG (mmol/L)	6.56±2.92	6.22±1.91	0.513
UA (μmol/L)	310.51±87.15	316.98±88.07	0.729
TC (mmol/L)	4.03±0.95	4.21±0.94	0.390
TG (mmol/L)	1.68±0.84	1.80±0.95	0.541
HDL-C (mmol/L)	1.38±0.26	1.45±0.47	0.428
LDL-C (mmol/L)	2.05±0.66	2.10±0.63	0.702
Lpa(mg/L)	240.08±15.54	217.91±207.66	0.623

mCAS, mild coronary artery stenosis; CAD, Coronary Artery Disease; CRP, C-reaction protein; PLT, Platelet; FIB, Fibrinogen; INR, International Normalized Ratio; BUN, Blood Urea Nitrogen; Cr, Creatinine; FBG,Fasting Blood Glucose; UA, Uric Acid; TC,Total Cholesterol; TG, Triglyceride; HDL-C, High Density Lipoprotein-cholesterol; LDL-C, Low Density Lipoprotein-cholesterol; Lpa, Lipoprotein a.

* $P < 0.05$, & $P < 0.01$.

Supplemental Table 5.

Univariate analysis and multiple logistic regression analysis for the risk of **mCAS**.

**Models**	***OR***	***95%CI***	***P* value**
Univariate analysis	0.438	0.253-0.758	0.003[&]
Multiple logistic regression model[1]	0.494	0.275-0.886	0.018*
Multiple logistic regression model[2]	0.491	0.274-0.881	0.017*
Multiple logistic regression model[3]	0.499	0.274-0.908	0.023*
Multiple logistic regression model[4]	0.439	0.223-0.864	0.017*
Multiple logistic regression model[5]	0.462	0.223-0.957	0.038*
Multiple logistic regression model[6]	0.170	0.056-0.518	0.002[&]

OR, odds ratio; CI, confidence interval.

The model[1] included age, gender, smoking, and SOCS2-AS1 level; The model[2] included age, gender, smoking, hypertension, and SOCS2-AS1 level; The model[3] included age, gender, smoking, hypertension, diabetes, FBG, and SOCS2-AS1 level; The model[4] included age, gender, smoking, hypertension, diabetes, FBG, BUN, Cr, UA, and SOCS2-AS1 level; The model[5] included age, gender, smoking, hypertension, diabetes, FBG, BUN, Cr, UA, CRP, PLT, FIB, INR and SOCS2-AS1 level; The model[6] included age, gender, smoking, hypertension, diabetes, FBG, BUN, Cr, UA, CRP, PLT, FIB, INR, TC, TG, HDL-C, LDL-C, Lpa, and SOCS2AS1 level.

* $P < 0.05$, & $P < 0.01$.
